# The Effect of Bacteria on the Stability of Microfluidic-Generated Water-in-Oil Droplet

**DOI:** 10.3390/mi13122067

**Published:** 2022-11-25

**Authors:** Nur Suaidah Mohd Isa, Hani El Kadri, Daniele Vigolo, Konstantinos Gkatzionis

**Affiliations:** 1 School of Chemical Engineering, University of Birmingham, Birmingham B15 2TT, UK; 2 Faculty of Fisheries and Food Science, Universiti Malaysia Terengganu, Kuala Terengganu 21030, Terengganu, Malaysia; 3 School of Biomedical Engineering, The University of Sydney, Sydney, NSW 2006, Australia; 4 The University of Sydney Nano Institute, The University of Sydney, Sydney, NSW 2006, Australia; 5 Department of Food Science and Nutrition, University of the Aegean, Metropolite Ioakeim 2, 81400 Myrina, Lemnos, Greece

**Keywords:** microfluidics, droplet stability, W/O droplets, microencapsulation, physiological status

## Abstract

Microencapsulation in emulsion droplets has great potential for various applications such as food which require formation of highly stable emulsions. Bacterial-emulsion interactions affect the physiological status of bacteria while bacterial cell characteristics such as surface-active properties and metabolic activity can affect emulsion stability. In this study, the viability and growth of two different bacterial species, Gram-negative *Escherichia coli* and Gram-positive *Lactobacillus paracasei*, encapsulated in water-in-oil (W/O) droplets or as planktonic cells, were monitored and their effect on droplet stability was determined. Microencapsulation of bacteria in W/O droplets with growth media or water was achieved by using a flow-focusing microfluidic device to ensure the production of highly monodispersed droplets. Stability of W/O droplets was monitored during 5 days of storage. Fluorescence microscopy was used to observe bacterial growth behaviour. Encapsulated cells showed different growth to planktonic cells. Encapsulated *E. coli* grew faster initially followed by a decline in viability while encapsulated *L. paracasei* showed a slow gradual growth throughout storage. The presence of bacteria increased droplet stability and a higher number of dead cells was found to provide better stability due to high affinity towards the interface. The stability of the droplets is also species dependent, with *E. coli* providing better stability as compared to *Lactobacillus paracasei*.

## 1. Introduction

Bacterial microencapsulation within emulsion droplets has been extensively exploited for various applications such as to increase bacteria viability in food products, to protect bacteria against the harsh conditions in the gastrointestinal tract and for high-throughput bacterial studies [1,2,3,4,5]. Emulsion droplets provide a gentle encapsulation for sensitive bacteria and improve their viability [3]. The most common method is by encapsulating bacteria in single (W/O) emulsion droplets [5,6,7] while bacterial encapsulation in multiple emulsion droplets has also gained interest, allowing for bacterial compartmentalization and controlled release [8,9,10,11].

The successful applications of emulsion droplets for bacterial encapsulation in food products are highly dependent on their stability during processing, storage and up to the point of consumption. Understanding the effects of the encapsulated bacteria on the emulsion stability is still limited and therefore requires further studies as bacterial response such as growth, death and production of by-products may affect stability. Previous studies revealed the potential use of bacteria for emulsion stabilization where bacterial surface properties played a key role in stabilization [12,13,14]. However, the mechanisms relating droplet stability and the presence of bacterial cells is highly complex and thus further studies may provide beneficial information in order to clearly understand the factors involved in this process.

Therefore, this study aimed to investigate the interrelationship between bacterial response and emulsion stability under a precisely controlled environment (i.e., droplet size and cell encapsulation) achieved with the aid of microfluidics. The stability of model emulsion systems in the presence of *Escherichia coli* (*E. coli*), the most common Gram-negative bacteria used in biotechnology applications such as in vitro synthesis of biomolecules [15], and *Lactobacillus paracasei* (*L. paracasei*) which is one of the most common Gram-positive bacteria used in the food industry for making dairy products such as yoghurt, were investigated during storage. The viability of the bacteria and their effect on droplet stability was investigated by measuring changes in droplet size distribution during storage and by characterising factors that affect stability through bacterial hydrophobicity and zeta potential tests. The developed model emulsion system and the study of bacterial interactions provide insights into the stability of such systems that can later be applied in the industrial production of emulsion-based products.

## 2. Materials and Methods

### 2.1. Materials and Bacterial Cultures

Microfluidic device fabrication was carried out by using a polydimethylsiloxane (PDMS) preparation set (Sylgard 184, Dow-corning, Midland, MI, USA) which includes the curing agent and the polymer base. Oil-soluble surfactant, polyglycerol polyricinoleate (PGPR) was obtained from Danisco while mineral oil and acridine orange stain (AO) were purchased from Sigma Aldrich (St. Louis, MO, USA). For bacterial culture preparation, the materials used were nutrient agar, De Man, Rogosa and Sharpe (MRS) agar and broth, Luria Bertani broth (LB broth) and phosphate buffered saline (PBS) all by Oxoid Ltd. (Basingstoke, UK). Propidium iodide stain (PI) was purchased from Invitrogen. *Escherichia coli* (*E. coli*) expressing green fluorescent protein strain SCC1 (MG1655-GFP mutation) and *Lactobacillus paracasei* subsp. *paracasei* DC412 (*L. paracasei*) stock cultures were obtained from the Biochemical Engineering Laboratory, University of Birmingham, United Kingdom.

### 2.2. Microfluidic Device Fabrication

**Figure 1:**
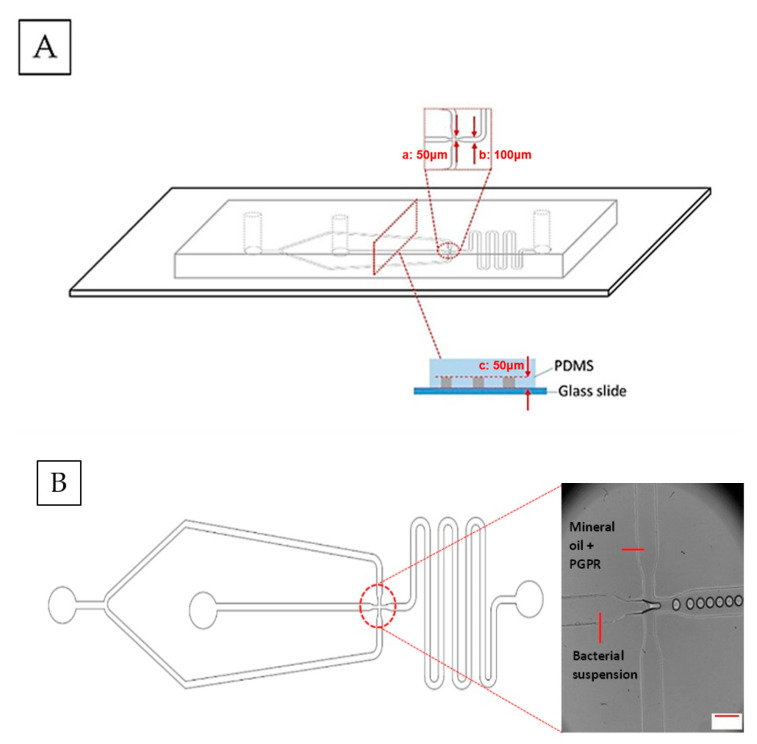
(**A**) Flow-focusing microfluidic device fabrication for W/O droplet generation: width at the junction (a): 50 µm, width at the exit channel (b): 100 µm and depth (c): 50 µm. (**B**) Monodispersed W/O droplet formation whereby W/O droplets were formed at the flow-focusing junction (in the circle). Scale bar represents 100 µm.

The microfluidic devices were fabricated by following standard soft lithography techniques [16]. The devices were designed according to Bauer et al. [17] using AutoCAD 2016 (Autodesk) software. The design was then printed onto high-resolution photo-masks (Micro Lithography Services Ltd., Chelmsford, UK). A patterned mould was produced by exposing a silicon wafer (Si-Mat) that was spin-coated with SU-8 photoresist (SU-8, Microchem) to UV light through the photomasks with a mask aligner. The devices were then prepared by mixing the PDMS and curing agent at the recommended mixing ratio of 10:1. The prepared PDMS was then poured onto the mould, degassed and cured in an oven at 70 °C for at least 1 h. The devices were then cut out of the mould and the inlet and outlet holes were created using a biopsy punch, followed by corona discharge treatment (PZ2 Handheld Device, Relyon Plasma GmbH, Regensburg, Germany) for approximately 30 s to bond the devices onto a glass slide to close the channels. The prepared devices were then left on a hot plate for approximately 15 min at 100 °C. A new device was prepared for every experiment in order to minimize contamination. A flow focusing device, with a typical width at the junction of 50 µm and of 100 µm width at the exit channel, and of 50 µm (depth), was used for producing water-in-oil (W/O) droplets with diameters between 40 and 50 µm (Figure 1).

### 2.3. Bacterial Cells Preparation

Bacterial cultures for encapsulation in W/O emulsion were prepared by culturing *E. coli* on nutrient agar at 37 °C for 24 h. The cultured bacteria were kept at 4 °C prior to the experiment. The bacterial cells were then inoculated into 50 mL of Luria Bertani (LB) broth and incubated in a shaking incubator at 37 °C, 150 rpm for 24 h. The cells were then sub-cultured into 50 mL of LB broth and incubated for another 2 h. The bacterial culture was then centrifuged (10,000× *g*, 10 min) and washed two times with 50 mL of PBS. After centrifugation, the supernatant was discarded and was replaced with 50 mL of fresh LB broth or sterilised DIW to re-suspend bacterial cells for encapsulation. The bacterial cells concentration was prepared to 10^8^ CFU/mL by serially diluting the stock culture until the desired cell concentration was achieved. *L. paracasei* culture was maintained on MRS agar at 4 °C. The cells were inoculated into 50 mL of MRS broth and incubated for 48 h at 25 °C, sub-cultured into 50 mL of fresh MRS broth and incubated for another 12 h. After incubation, approximately 10^8^ CFU/mL of *L. paracasei* cells were obtained and 50 mL of cell suspension was centrifuged at 10000 × g for 10 min. The cells were washed twice with PBS and re-suspended into 50 mL MRS broth or sterilised deionized water for encapsulation into W/O droplets.

Samples of dead cells were prepared for determining the characteristics of both live and dead cells by suspending bacterial cultures of 10^8^ CFU/mL in sterilised DIW and heat-treating at 80 °C for 30 min using a thermomixer (Eppendorf). 80 °C is the temperature used in pasteurization that resulted in the total reduction of viability for many bacterial strains including *E. coli* and *Lactobacillus* strains [18]. The heat-treatment of cells may result in dead cells and viable-but-not-culturable (VBNC) cells that were extensively injured to the point where they were unable to grow on nutrient agar (for *E. coli*) and MRS agar (for *L. paracasei*). In this study, the viability of bacteria was determined based on membrane integrity; thus, a bacterial cell with the inability to grow on agar plates together with positive staining of PI was termed as a dead cell.

### 2.4. Bacterial Encapsulation in Single Water-in-Oil Emulsion

*E. coli* in LB broth and *L. paracasei* in MRS broth were then used as the aqueous phase of the W/O droplet with 10^8^ CFU/mL of cell concentration. The continuous oil phase consisted of mineral oil with 1.5% *w*/*v* PGPR surfactant. The bacterial encapsulation was conducted by using a flow-focusing microfluidic device (Figure 1). The flow was controlled by syringe pumps (AL-1000, World Precision Instruments, Sarasota, FL, USA) and the flow rates used for droplet production were 3/µL min for the dispersed aqueous phase containing bacteria and 30/µL min for the continuous oil phase, forming droplets of approximately 40–50 µm in diameter (Figure 1). W/O droplets with *E. coli* or *L. paracasei* in DIW as the aqueous phase were used as non-nutrient controls. In addition, empty droplets of LB broth, MRS broth and DIW were also produced as controls to determine the effects of bacteria on droplet stability. All samples were kept statically in Eppendorf tubes at 25 °C for five days.

### 2.5. Determination of Bacterial Viability

Viability was determined for bacteria encapsulated in W/O droplet with or without nutrient. Unencapsulated bacteria dispersed in sterilised DIW or LB broth (for *E. coli*) and MRS broth (for *L. paracasei*) were prepared as controls. Encapsulated samples were centrifuged at 15,800× *g* for 10 min in order to break the emulsion and release the entrapped cells. Viable cells were counted daily for both encapsulated and unencapsulated samples during the five days of storage using the Miles and Misra method [19]. Serial dilutions were done on the sample with PBS. For *E. coli*, 10 µL of diluted sample was pipetted onto nutrient agar and incubated at 37 °C for 24 h while for *L. paracasei*, samples were pipetted onto MRS agar and incubated at 25 °C for 48 h.

### 2.6. Optical Microscopy for Determining Droplet Stability Changes during Storage

Droplet size was measured daily for all W/O emulsion samples during the five days of storage at 25 °C. In order to measure the size of the droplet, a drop of the W/O sample was placed on a glass slide, covered with a coverslip and observed at 10× magnification by using a Nikon Eclipse Ti-U microscope. Images of the droplets were taken (N = 900) and analysed by using MATLAB (MathWorks, Natick, MA, USA) software for size measurement using the circular Hough transform [20]. For characterisation of droplet size distribution, changes in the coefficient of variation (CV) were determined by dividing the standard deviation by the mean of droplet size [21,22,23].

### 2.7. Fluorescence Microscopy for Bacterial Response Observation

Fluorescence microscopy was conducted in order to establish the viability of the encapsulated bacteria in W/O emulsion droplets. Dead cell observation was performed by staining *E. coli* and *L. paracasei* with PI. Additionally, *L. paracasei* were stained with AO for viable cell observation.

The samples were prepared for microscopy by placing 1 drop of sample onto a glass slide and covering it with a coverslip. The samples were then observed using a microscope (Zeiss Axioplan, Oberkochen, Germany) through a 100× immersion oil objective and micrographs of the samples were acquired using the Axiocam ICm1 (Zeiss) digital camera system (1.4-megapixel) and Axiovision software (Zeiss). The emission was observed at 509 nm (GFP), 502 nm (AO) and 645 nm (PI) using a mercury arc lamp. The micrographs obtained were overlaid and image analysis was done using ImageJ to determine the size of bacterial clustering with respect to droplet size.

### 2.8. Observation of Bacterial Clustering with Confocal Microscopy

In order to clearly observe the clustering of bacteria in the W/O droplet, images of *E. coli* clustering in the droplet containing LB Broth were taken using a Leica TCS SPE confocal scanning microscope at 100× magnification. GFP excitation was observed at 509 nm. Images were taken at 3 µm intervals over the whole droplet thickness to provide clear observation of the overall 3D structure of bacterial clustering within the W/O droplet.

### 2.9. Bacterial Hydrophobicity Test

Bacterial hydrophobicity of live and dead cells of *E. coli* and *L. paracasei* was tested. The assay was conducted according to Rosenberg et al. [24]. Washed bacterial cells (1.2 mL of live or dead) in sterilised DIW were added into four round bottom test tubes. Different volumes of mineral oil (0.2, 0.15, 0.1, 0.05 mL) were then added into the test tubes. After 10 min of incubation at room temperature, the samples were mixed by vortexing the test tubes for 2 min. After mixing, the samples were left upright at room temperature to allow the separation of the oil and bacterial suspension. The aqueous phase was then carefully drawn out of the test tubes by using a pipette and the absorbance of the aqueous phase was measured at 600 nm for the samples after being mixed with mineral oil. The percentage of absorbance was calculated for samples after treatment relative to samples before treatment with different volumes of mineral oil.

### 2.10. Interfacial Tension Determination between Bacteria and Mineral Oil

The interfacial tension between the bacterial suspension and mineral oil was measured by the pendant drop method using an Attension Theta optical tensiometer (Biolin Scientific, Stockholm, Stockholms Lan, Sweden) and comparisons were made between live and dead bacterial cells suspended in sterilised deionised water (DIW) against mineral oil (with or without 1.5% PGPR). Live and dead cells were prepared at different concentrations of 10^1^, 10^3^, 10^6^, 10^8^ CFU/mL with OD600 values of 0.07, 0.4, 0.7, and 2.3, respectively. Samples consisting of DIW without bacteria were used as control. The samples were prepared by serial dilutions and the OD_600_ were measured by using a spectrophotometer (Jenway 6305, Bibby Scientific Ltd., Stone, UK) to determine the effect of different bacterial concentrations on interfacial tension. Samples containing a mixture of live and dead cells suspended in DIW at different ratios (Live: Dead, 30:70, 50:50, 70:30) were also tested. A drop of the sample (11 µL) was introduced into the mineral oil and was left to stabilise for 3 min. Interfacial tension readings (mN/m) were then measured using the software OneAttension (Biolin Scientific, Stockholm, Stockholms Lan, Sweden) and the process was repeated for 3 droplet replicates.

### 2.11. Bacterial Surface Zeta Potential Determination

The zeta potential of the bacterial suspensions was measured for both live and dead cells of *E. coli* and *L. paracasei* in different suspension solutions to determine colloidal stability. Samples were prepared by suspending live or dead cells in different suspensions (LB broth for *E. coli*, MRS for *L. paracasei* or DIW). In addition, mixed samples containing both live and dead cells suspended in DIW with different ratios (L:D, 70:30, 50:50, 30:70) were also prepared. Approximately 1 mL of diluted sample (OD_600_ = 0.34) was carefully loaded into a folded capillary zeta cell and the zeta potential was measured using Zetasizer Nano ZS by Malvern Instruments (Malvern, UK).

### 2.12. Statistical Analysis

The experiments were conducted with three replicates. For the experiment that determines the effect of variables on droplet diameter, a total of 900 droplets were measured (N = 900). The generated data were analysed with Excel (Microsoft Corp., Redmond, MA, USA) to calculate the mean, standard deviation (SD), standard error of the mean (SEM) and coefficient of variation (CV). Student’s *t*-test was conducted in order to compare two means while one-way ANOVA with Tukey’s HSD was conducted to compare several means by using IBM SPSS statistical software version 21. The difference between the means was considered significant at *p* < 0.05.

## 3. Results and Discussion

### 3.1. The Effect of Bacteria on Droplet Stability

**Figure 2:**
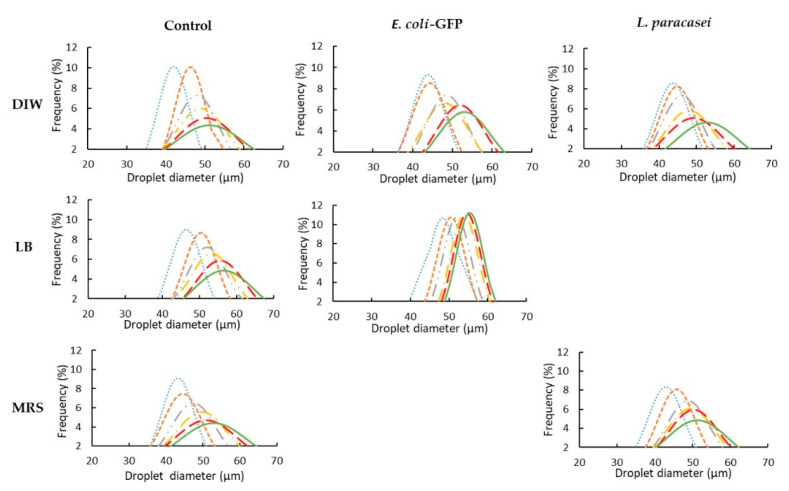
Droplet size distribution at Day 0: blue (····), Day 1: orange (----), Day 2: grey (- ··), Day 3: yellow (- · -), Day 4: red (- - -), Day 5: green (⸺). Frequency (%) refers to the percentage of droplets. Data was analysed with N = 900 droplets.

In order to understand the effects of bacteria on the stability of W/O emulsions, changes in droplet size distribution during storage of bacteria (*E. coli* or *L. paracasei*) encapsulated with nutrient or DIW were monitored (Figure 2). The droplet size distribution was characterized by measuring the CV whereby a CV below 25% indicates a monodisperse droplet while emulsions with CV above 25% are regarded as polydispersed [23].

From the results obtained, it was observed that at day 0, the measured diameter of the droplet generated by the microfluidic device for all samples was approximately 40–50 µm and monodispersed droplets were produced for all samples indicating the ability of the designed flow-focusing microfluidic device to produce monodispersed droplets (Figure 2). Due to the monodispersity of the droplets formed at day 0 for all samples, the polydispersity effects were excluded in determining the stability of the droplet.

In general, the average droplet size for all the samples tested increased after five days of static storage at 25 °C (Table 1). A significant increase (*p* < 0.05) in droplet diameter was observed immediately after one day of storage for all the control samples of empty droplets (empty DIW, LB broth and MRS broth). However, for samples containing bacteria (*E. coli* in DIW, *E. coli* in LB broth, *L. paracasei* in DIW and *L. paracasei* in MRS broth), a significant increase (*p* < 0.05) in droplet diameter were only observed after two days of storage. Comparing the overall diameter changes (%) between the empty droplet of DIW, LB and MRS broth shows that the addition of nutrient of both LB and MRS did not significantly affect (*p* > 0.05) the changes in droplet size. Moreover, the addition of *E. coli* in LB broth and *L. paracasei* in MRS broth resulted in a significantly lower percentage of change (*p* < 0.05) in the overall droplet diameter when compared to control (LB and MRS broth).

The results obtained (Table 1) showed that all samples remained monodispersed during five days of storage due to the presence of PGPR as surfactants in the continuous oil phase. Although the monodispersity of samples was maintained during storage, a significant increase in CV value was observed during five days of storage for all the control samples of empty droplet with or without nutrients. The presence of *E. coli* in LB broth, resulted to only 1.3% changes in CV value after five days of storage while the empty LB broth samples showed an increase in CV value of 5.5%. The same trend was observed for *E. coli* in DIW whereby 3.2% and 8.7% increase in CV value was observed for droplets with bacteria and empty DIW droplets, respectively. Samples containing *E. coli* showed a smaller change in CV value as compared to *L. paracasei*, whereby samples containing *L. paracasei* in DIW showed a 5.5% increase in CV value as compared to *E. coli* with only 3.2% increase. The results in Table 1 strongly suggest the role of bacteria in droplet stabilization which is species dependent as *E. coli* appears to stabilize the emulsion better than *L. paracasei*. Droplets containing bacteria encapsulated with nutrient had better stability as compared to encapsulation in water for both *E. coli* and *L. paracasei*. The increase in the average droplet size and CV value for control samples of empty droplet after five days of storage indicates instability due to the occurrence of emulsion breakage and coalescence during storage which was minimized in samples containing bacterial cells (Table 1).

It has been reported previously that bacterial cells may act as Pickering particles that help in the stabilization of O/W emulsions [12,13,14] which explains the stability of the droplets incorporated with bacteria. The encapsulated bacteria in W/O droplet may act as particles that aid in maintaining the stability of the droplet. The stabilization effect of “bacteria particles” in the emulsion is due to the formation of Pickering emulsions whereby the adherence of bacterial cells onto the interface helps in reducing the interfacial tension. Furthermore, the addition of nutrients that promote the growth of bacterial cells also plays an important role in droplet stabilization as the encapsulation of bacteria with nutrients increases the number of cells in the droplet that act as particles, thus, improving bacterial coverage on the interface of the droplet.

The results strongly suggest the effect of bacterial cells in droplet stability. However, it does not result in permanent stabilization as droplet size was increased after five days of storage. This may be attributed to the active coarsening that occurs between droplets containing bacteria and empty droplets within the same system. This has been observed in previous studies of bacteria encapsulated in single W/O emulsion [25,26,27]. In a single emulsion system containing bacteria as the aqueous phase, active coarsening of the droplet is driven by the osmotic imbalances between droplet containing bacteria and empty droplets within the same emulsion system. Nutrient depletion due to bacterial bioactivity reduces the overall solute concentration within the droplet causing osmotically driven water flux from droplets containing bacteria to neighbouring empty droplets. This causes the droplets containing bacteria to shrink while the empty droplets swell [25,26,27]. The mixture of empty and occupied droplets creates an osmotically imbalanced environment between droplets that may cause a shift in droplet distribution towards the larger-sized droplets (Figure 2).

Poisson statistics have been used previously to predict droplets occupancy; cells originated from the initial suspension were distributed according to the Poisson distribution, and the growth of bacteria within the droplets has been associated with droplet shrinkage [27]. However, as droplets shrinkage was driven by the difference in solute concentration between the droplets, factors such as NaCl concentration within the occupied droplets—as it was not being consumed during bacterial growth—and the production of metabolites may affect the overall droplet shrinkage or swelling. In addition, it may also be affected by the proximity of the empty and occupied droplets as when an empty droplet is surrounded by occupied droplets, this is more likely to assist water flux between the droplets that increases droplet swelling and vice versa [27]. However, further studies are still required to clearly understand the relationship between droplets occupancy and droplets size change.

### 3.2. The Viability of Encapsulated Bacteria during Storage

**Figure 3:**
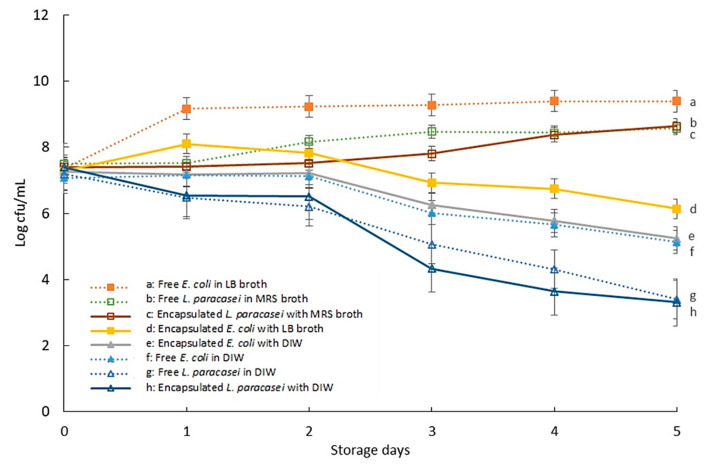
Bacterial growth during five days of storage for free bacterial cells and encapsulated bacterial cells in W/O droplet with or without nutrients. Bars represent mean ± SEM taken from 3 independent experiments (N = 3) with 30 µL of sample tested with Miles and Misra technique for each experiment.

The viability of bacterial cells was determined to study the effect of encapsulation on bacterial growth as presented in Figure 3. As expected, a decrease in viable cells was observed for both *E. coli* and *L. paracasei* suspended in DIW. Encapsulation in W/O droplets with DIW did not improve the viability of bacterial cells as a decrease in viable cells was also observed. This is due to the lack of nutrient that had caused the cells to enter the death phase. Referring to samples of *E. coli* suspended in LB broth, an increase in the growth of bacterial cells was observed for free *E. coli* cells during storage while an increase in the growth of *E. coli* cells encapsulated in W/O droplet was only observed on the first day of storage. This shows that encapsulation in W/O droplets inhibited the growth of *E. coli*. However, *L. paracasei* suspended in MRS broth shows increase in growth for both encapsulated and non-encapsulated samples during the incubation period.

The difference in viability between *E. coli* and *L. paracasei* may be attributed to the difference in growth rate as *E. coli* has a higher growth rate as compared to *L. paracasei*. The generation time, which is the amount of time required by bacteria cells to double in number, is reported to be around 20 min for free *E. coli* cells in LB broth under optimum conditions [28] and one hour for *lactobacilli* [29,30,31]. The rapid growth of *E. coli* cells during the first day of storage speeds up nutrient depletion in each W/O droplet resulting in a decrease in bacterial viability after one day of incubation due to its inability to support the growth of bacteria in the droplet. This is in contrast with samples containing *L. paracasei* whereby a slower growth rate had caused slower depletion in nutrients and therefore helped in maintaining the growth of *L. paracasei* during five days of storage. Encapsulation limits the availability of nutrients and space for bacterial growth, which lowers the bacterial growth rate and yield. Similar behaviour has been previously reported in the study of bacterial growth in O/W emulsion whereby inclusion of bacteria in the crowded environment of oil droplets reduced the growth rate and yield of bacterial cells [32]. In that study, the growth of bacteria was inhibited from planktonic to clustering that resulted in a reduced growth rate. In addition, the accumulation of bacterial metabolic end products may also inhibit the growth of encapsulated bacteria. Moreover, the low oxygen permeability of mineral oil as compared to water limits the diffusivity of oxygen for maximum bacterial growth.

Nevertheless, referring to the droplet size distribution result (Figure 2), the stability of the droplet was highly maintained for droplets containing bacteria even though the bacterial viability showed a decrease during storage. This was also true for bacteria encapsulated in DIW which improved the stability of droplet although a decrease in cell viability was observed. The decrease in bacterial viability indicates the presence of dead cells within the droplet. Therefore, the stability of droplet with reduced cell viability indicated the role of dead cells in droplet stabilization.

### 3.3. Microscopic Observation of Bacterial Response in W/O Droplet

Following the results obtained from droplet stability and bacterial viability tests, it was hypothesized that the inclusion of bacterial cells in the W/O droplet promotes droplet stability during storage. In addition, droplet stability was also attributed to the presence of dead cells within the droplet during five days of incubation. To provide a clear explanation of the mechanism of droplet stability due to bacteria, a microscopic observation was conducted during the incubation period to distinguish the growth of bacteria and the presence of dead cells within the droplet.

**Figure 4:**
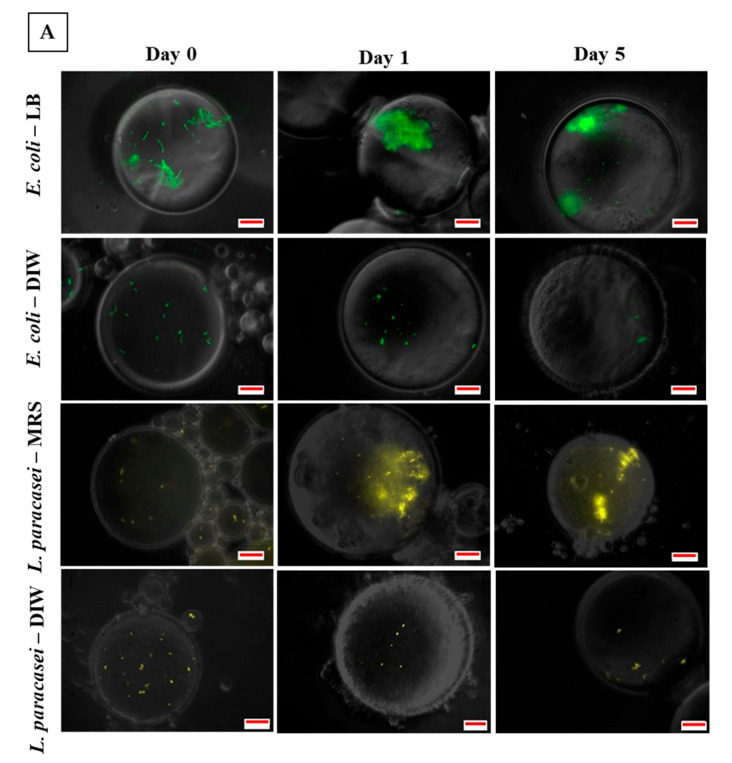

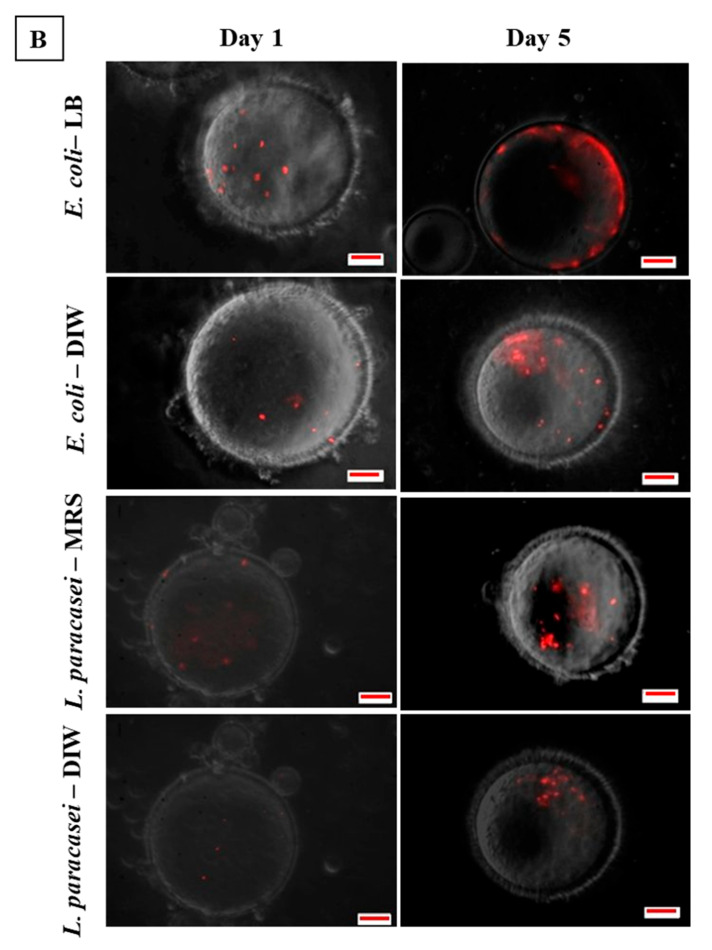
Photomicrographs of bacterial cells encapsulated in W/O droplets during storage showing (**A**) live cells and (**B**) dead cells. Bacterial clusters were observed during storage for samples encapsulated with nutrient. Presence of dead cells were also observed after one day of storage for all samples of bacteria encapsulated with or without nutrient. Colour coding—Green: Live *E. coli* cells, yellow: Live *L. paracasei* cells and red: Dead cells. Scale bar: 10 µm.

**Figure 5:**
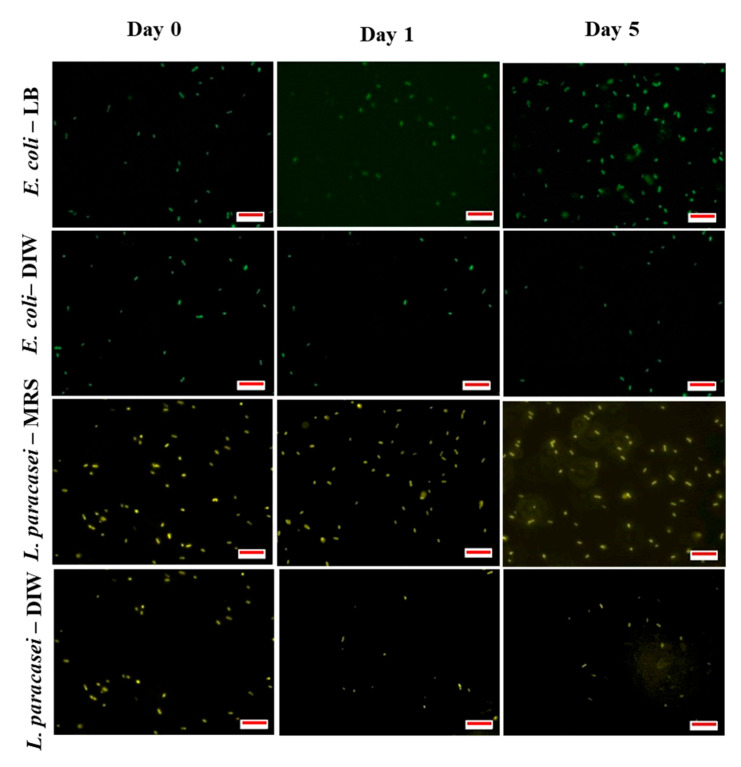
Photomicrographs of free (unencapsulated) bacterial cells suspended in LB broth (for *E. coli*, shown by green coloured cells) and MRS broth (for *L. paracasei,* shown by yellow coloured cells). No bacterial clusters were observed during five days of storage. Scale bar: 10 µm.

From the photomicrographs of fluorescence microscopy for encapsulated *E. coli* samples (Figure 4a), rope-like structures were observed after two hours (at day 0) of bacterial encapsulation in LB broth that leads to the formation of bacterial clustering after one day of storage. The formation of bacterial clustering was only observed for bacteria encapsulated with LB broth while droplets containing bacteria in DIW (Figure 4a) and unencapsulated samples in DIW or LB broth (Figure 5) remained planktonic. For *L. paracasei* samples, the presence of bacterial clusters was only observed after five days of storage for samples encapsulated with nutrient and no bacterial clusters were observed for samples encapsulated with DIW (Figure 4a) or unencapsulated samples in DIW or MRS broth (Figure 5). This is in agreement with studies done by Chang et al. [25] and Barlow et al. [33] whereby clusters of bacteria were observed when biofilm-forming *Bacillus subtilis* was encapsulated in a single W/O emulsion and in the inner phase of double W_1_/O/W_2_ emulsion. As expected, the presence of dead cells was observed after one day of storage (Figure 4b).

In the study done by Chang et al. [25] on the growth of biofilm in a microfluidic-generated droplet, clumps of bacteria were observed in W/O droplets containing *Bacillus subtilis*. However, due to the absence of a film-former surfactants such as silicone that can provide a substrate for biofilm adhesion, the bacterial biofilm was not formed on the interface of the droplet but was seen floating in the aqueous phase similar to the results obtained by Barlow et al. [33]. It was reported that with a continuous supply of nutrients from the outer aqueous phase of double W_1_/O/W_2_ emulsion, rapid formation of biofilm was observed as early as four hours of *Bacillus subtilis* encapsulation with the formation of rope like structures [33]. In this study, the onset of biofilm formation by *E. coli* in W/O droplet was demonstrated with the formation of rope-like structures after two hours of encapsulation that grew into distinct bacterial clusters after one day of storage. This is mainly due to the high growth rate of *E. coli*. Environmental stress such as nutrient depletion, lack of oxygen and space for growth leads to several morphological and physiological changes in microorganisms. In such conditions, *E. coli* responded by ceasing all metabolic activity and growth in order to prolong their survival [34]. Stress-induced enzymes were produced along with the accumulation of several storage compounds such as glycogen and polyphosphate. In addition, changes in cell size and shape were also observed which may lead to the formation of bacterial biofilms [34,35]. However, as observed in this study and reported previously, the formation of bacterial clusters and biofilms for lactobacilli strains is less distinct as the limitation in growth due to environmental stress such as lack of nutrients was not sufficient to induce the formation of biofilm [36].

**Figure 6:**
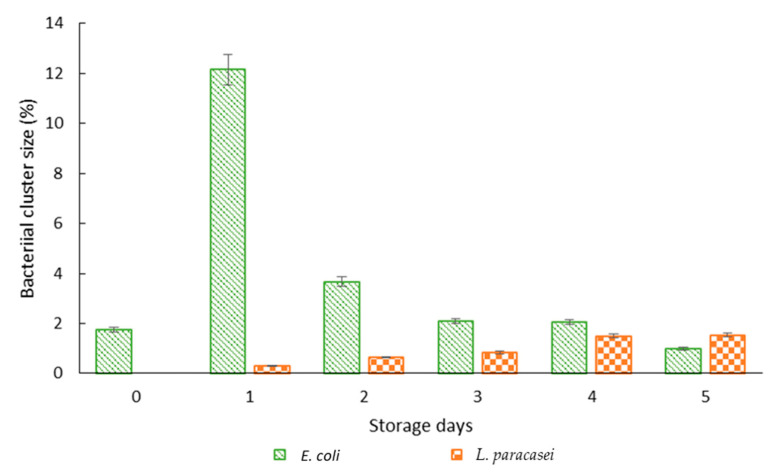
Cluster size with respect to storage days. Bars represent mean ± SEM taken from 3 independent experiments. A total of 10 droplets were measured for each experiment (N = 10).

**Figure 7:**
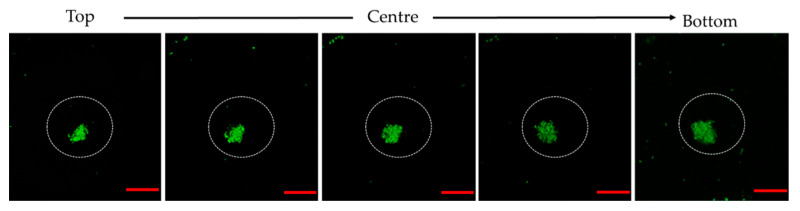
Cross-sectional images of *E. coli* clustering cells taken using a confocal microscope at day one of storage. Scale bar: 25 µm.

To get a clear view of the formation of bacterial clusters, the percentage and size of bacterial clustering with respect to the area of the droplet was quantified by analysing fluorescence photomicrographs with ImageJ (Figure 6). In addition, confocal microscopy of the encapsulated *E. coli* was also done after one day of storage as presented in Figure 7. For *E. coli*, a large percentage of the bacterial cluster was observed on day one which started to decrease after two days of storage as opposed to *L. paracasei* whereby smaller cluster size was formed throughout the storage period. Day 0 shows a relatively small percentage of bacterial clustering for *E. coli* due to the formation of rope-like structures at the beginning of the storage period. A decrease in bacterial clusters for *E. coli* after 2 days of storage was in response to nutrient depletion [37]. In addition, the lack of surface for attachment eased the process of cell detachment. The presence of planktonic dead cells was observed after 1 day of storage with the majority seen at the interface indicating the ability of dead bacterial cells to act as Pickering particles for droplet stabilization (Figure 4b). Due to distinct bacterial clusters formed after one day of storage, confocal microscopy was done on samples containing *E. coli* that produced a 3D image of the droplet (Figure 7). Cross-sectional images of bacterial clustering show the formation of bacterial clustering that extends from the top to the bottom of the droplet.

### 3.4. Characterisation of Bacterial Hydrophobicity

Bacterial cells were characterised in terms of hydrophobicity by measuring absorbance before and after mixing with mineral oil and water to determine percentage cell adherence to mineral oil. A decrease in absorbance with an increase in mineral oil volume for dead cells was observed while the presence of live cells resulted in no changes in the absorbance (Figure S1). The decrease in absorbance indicated an increase in the overall hydrophobicity as more dead bacterial cells adhere to the mineral oil. Comparing between *E. coli* and *L. paracasei*, it was observed that dead *E. coli* cells exhibited better affinity towards mineral oil compared to dead *L. paracasei*. This is attributed to the difference in lipid and lipoprotein content of the bacteria whereby the Gram-negative *E. coli* has higher lipid content as compared to the Gram-positive *L. paracasei,* due to the presence of the outer membrane in the Gram-negative bacteria [38].

The results obtained provided an explanation on the stabilization mechanism of bacterial cells incorporated in W/O emulsion droplet. At the beginning of the storage time, an increase in bacterial cells occurred that leads to bacterial clustering. During this time, droplet stability was mainly maintained by the presence of surfactants at the interface as live cells with weak hydrophobicity prefer to stay in the aqueous phase rather than at the interface. After one day of storage, the presence of dead cells with high affinity towards the oil phase was observed that aided in maintaining droplet stability. It has been reported previously that thermally-inactivated microbial cells may act as particles in stabilizing Pickering oil-in-water emulsions via hydrophobic interactions, whereby the denaturation of proteins in the bacterial cell wall caused the exposure of hydrophobic groups [14]. The ability of bacteria to adsorb onto the water-oil interface depends on several factors such as cell surface characteristics, size and bacterial concentration. Nevertheless, changes in bacterial characteristics such as cell size and shape along with changes in the fatty acid and protein composition of the bacterial membrane due to environmental stress, such as starvation, may contribute to the increase in bacterial affinity towards the interface [34]. In addition, the existence of pili on the bacteria which are known to assist its attachment onto surfaces and formation of biofilm may also play a role in the adsorption of bacteria onto the interface [14].

### 3.5. Changes in the Interfacial Tension of Droplets in the Presence of Bacteria

The interfacial tension was reduced with the addition of bacteria even at low concentration for both samples with or without PGPR (Supplementary Figure S2). It was shown that the interfacial tension was affected by bacterial concentration for both *E. coli* and *L. paracasei* samples (OD_600_ values of 0.07, 0.4, 0.7, and 2.3), whereby a reduction in interfacial tension was observed with an increase in bacterial concentration. The interfacial tension was better reduced in the presence of *E. coli* cells as compared to *L. paracasei* cells for both samples of live and dead cells with or without PGPR surfactant. As an example, in samples without PGPR surfactant, a reduction in interfacial tension was observed from 44.5 mN/m to 39.5 mN/m (5 mN/m reduction) as live *E. coli* cells increased to OD 0.4 whereas the interfacial tension was only reduced from 50.3 mN/m to 48.2 mN/m (2.1 mN/m reduction) for live *L. paracasei* cells (Figure S2a). It was determined that the addition of dead cells showed a better reduction in interfacial tension as compared to live cells for both samples of *E. coli* and *L. paracasei,* which may be attributed to the hydrophobic characteristic of the dead cells. Moreover, the addition of dead *E. coli* cells resulted in a better reduction of the interfacial tension as compared to dead *L. paracasei* due to the difference in lipid content between the two bacterial strains.

The surface-active effect of samples containing a mixture of both live and dead cells at different ratios (L:D, 30:70, 50:50, and 70:30) was also determined as shown in Supplementary Figure S3 which clearly shows the effect of dead cells in reducing interfacial tension. From the results obtained, reduction in interfacial tension was affected by the ratio of dead and live cells in the sample; samples containing a higher ratio of dead cells showed better surface-active characteristics due to better interfacial tension reduction as compared to samples containing a lower concentration of dead cells. A similar trend was observed in both systems with or without PGPR surfactant. This shows that dead cells play a major role in reducing the interfacial tension compared to live cells.

The effect of bacteria in reducing interfacial tension between the water and oil phase has been reported previously by Chen and Wang [39] whereby *E. coli* cells were shown to have a better effect on interfacial tension reduction as compared to *Chlorella vulgaris* (*C. vulgaris*) cells and polystyrene microparticles due to their higher affinity towards the hydrophobic interface. The ability of bacteria to improve emulsion stability is mostly attributed to bacterial surface properties whereby the wettability, bacterial size, shape and concentration play a major role in ensuring the effectiveness of the bacteria [12,14].

In addition, bacterial concentration is also important in ensuring effective coverage of the bacteria on the interface for stabilization. However, highly concentrated bacterial suspensions may cause the bacteria to cluster and diminish their ability to adhere to the interface. Therefore, further investigation into this matter may provide a better understanding on the mechanism of bacterial attachment onto the interface.

### 3.6. Colloidal Stability of Bacterial Suspension

The colloidal stability of samples containing bacterial cells in DIW or nutrient (LB or MRS broth) were determined by measuring the zeta potential of the suspension whereby values of more than +30 mV or less than −30 mV indicate a stable colloid. Colloidal instability is perceived at values between −30 mV to +30 mV as solute particles tend to flocculate [40]. In addition, the interactions between live and dead cells were also determined by testing mixed samples containing different ratios of live and dead cells.

From the results obtained, it was determined that bacterial samples suspended in LB or MRS broth have better colloidal stability as compared to samples in DIW. *E. coli* shows a reduction in zeta potential value from −19.8 mV to −34.6 mV for live cells and −26.48 to −36.36 mV for dead cells when suspended in LB broth. In addition, dead *E. coli* cells show lower zeta potential values as compared to live cells and an increase in the number of dead cells in mixed samples also causes a reduction in the zeta potential value (Supplementary Figure S4). This is in agreement with a study by Soni et al. [40] whereby a reduction in zeta potential value was observed for *E. coli* samples grown in the rich medium as compared to starved cells.

Changes in zeta potential were also observed when cells enter the death phase. This suggests that bacterial surface charge is affected by their nutrient state and their viability. The suspension of bacteria in LB or MRS broth along with changes in bacterial surface conformation as they enter the death phase also improve colloidal stability. For *L. paracasei*, the zeta potential value changed towards a positive value close to zero in MRS broth compared to DIW. This is probably due to the interactions between *L. paracasei* cells and other molecules in MRS broth whereby the presence of lactic acid due to fermentation of glucose in MRS broth lowered the pH and caused the carboxyl and phosphate groups on the cell’s surface to be protonated [41] and therefore improved colloidal stability. However, there is no difference observed in the zeta potential between live and dead *L. paracasei* samples and therefore changes in bacterial cell surface as they enter the death phase does not affect the surface charge and colloidal stability. This is also shown in samples containing mixed cells whereby an increase in the amount of dead cells does not affect changes in zeta potential (Supplementary Figure S4b).

The results obtained provide an overview of the stability of bacterial suspensions prior to droplet formation. The colloidal stability of bacterial suspensions is mainly due to the availability of nutrient in the suspension in which the interaction of bacteria with particulate solutes in nutrient (LB or MRS broth) improves colloidal stability. Other than that, the results show that for samples suspended in nutrient (LB or MRS broth), the colloidal stability of the samples indicates that the bacteria are more likely in the planktonic state rather than clustering.

## 4. Conclusions

The inclusion of bacterial cells in W/O droplets affected their stability. The Gram-negative *E. coli* showed better stabilization effects compared to the Gram-positive *L. paracasei*, which may be due to differences in lipid content. Further investigations revealed the ability of bacteria to act as Pickering particles in stabilizing the W/O droplet whereby better surface-active effects were shown by dead cells as compared to live cells. The interfacial studies of samples containing dead cells showed a higher reduction in interfacial tension as compared to samples containing live cells. The inability of bacteria to survive in W/O droplets during storage indicated the presence of dead cells with high affinity towards the interface (due to hydrophobic nature) which aids in maintaining the stability of the droplets during storage that was shown through the bacterial hydrophobicity test. The effects of bacteria on droplet stability depended on several factors such as the type of bacteria, bacterial viability and bacterial concentration; these factors play a major role in ensuring the effectiveness of bacteria as Pickering particles in promoting droplet stability.

Encapsulation of bacteria inside emulsion droplets can provide several advantages for various applications. For example, they can act as miniaturized bioreactors in the food or bioprocess technology, controlling the movement of nutrients from and into the droplets as well as segregating microbes during moromi fermentation to enhance flavor [10]. Furthermore, encapsulating bacteria in emulsion droplets has been shown to improve probiotic viability during yogurt fermentation [1] and during processing and gastrointestinal transit [42]. Encapsulation in droplets of controlled size allows for studying the behaviour of bacteria in confined spaces; for example, quorum sensing which normally occurs at higher cell density, was shown to occur at single cell level inside droplets [6]. In addition, biofilm formation can be studied at various stages in droplets under controlled environments [25]. However, none of these studies assessed the interrelationship between bacterial response and emulsion stability which is essential when designing an emulsion system for microbiological applications. Furthermore, the results obtained from this study provided an indication of the interrelationship between bacterial cells and W/O droplet microstructure that will be beneficial in understanding the stability of emulsion systems incorporated with bacteria. The proposed mechanism of bacterial stabilization effects in W/O droplet may be beneficial for the industrial application of emulsion systems with bacteria, for example, by segregating different microbial species during fermentation processes. In addition, it demonstrated the ability of droplet microfluidics in the development of model emulsion systems with controlled and monodispersed droplet size.

## Figures and Tables

**Table 1:** Summary of changes in droplet stability during five days of storage at 25 °C. Data represent the mean ± standard deviation from 3 independent experiments with N = 900 droplet. The average diameters were measured daily during five days of storage and the overall change in diameter (%) was measured based on the diameter at day 0 with respect to day 5. The CV values were measured by dividing the standard deviation by the average droplet diameter for each storage day. The average diameters at different storage days were compared within each sample while the overall diameter changes were compared between samples. Mean diameters with different letters indicate significant different (*p* < 0.05) between samples.

Samples	Days	Average Diameter (µm)	Overall Diameter Changes (%)	Coefficient of Variance (%)
Empty DIW	0	42.0 ± 3.9 ^a^	21.2 ± 10.7 ^a^	9.3
	1	46.3 ± 4.0 ^b^		8.6
	2	48.0 ± 5.5 ^bc^		11.5
	3	49.0 ± 6.7 ^cd^		13.6
	4	50.4 ± 7.9 ^d^		15.7
	5	51.2 ± 9.2 ^d^		18.0
Empty LB broth	0	46.5 ± 4.4 ^a^	21.3 ± 6.2 ^a^	9.5
	1	50.4 ± 4.6 ^b^		9.1
	2	52.1 ± 5.5 ^c^		10.6
	3	53.4 ± 6.0 ^cd^		11.2
	4	55.6 ± 6.8 ^de^		12.2
	5	56.6 ± 8.2 ^e^		14.5
*E. coli* in DIW	0	43.8 ± 4.3 ^a^	20.6 ± 3.9 ^a^	9.8
	1	44.3 ± 4.7 ^a^		10.6
	2	48.5 ± 5.4 ^b^		11.1
	3	48.1 ± 6.0 ^b^		12.5
	4	51.9 ± 6.2 ^c^		11.9
	5	53.0 ± 6.9 ^c^		13.0
*E. coli* in LB broth	0	48.3 ± 3.7 ^a^	14.8 ± 1.4 ^b^	7.7
	1	50.6 ± 3.7 ^ab^		7.3
	2	52.0 ± 3.7 ^bc^		7.1
	3	53.9 ± 3.6 ^cd^		6.7
	4	54.7 ± 3.6 ^d^		6.6
	5	55.4 ± 3.6 ^d^		6.4
Empty MRS broth	0	43.3 ± 4.4 ^a^	21.6 ± 8.7 ^a^	10.2
	1	44.6 ± 5.3 ^b^		11.5
	2	47.4 ± 6.1 ^b^		12.7
	3	49.7 ± 7.2 ^c^		14.5
	4	50.9 ± 8.5 ^c^		16.7
	5	52.9 ± 9.1 ^d^		17.3
*L. paracasei* in DIW	0	43.7 ± 4.7 ^a^	20.6 ± 6.8 ^ac^	10.8
	1	44.8 ± 4.8 ^ab^		10.7
	2	46.0 ± 5.4 ^b^		11.7
	3	48.1 ± 6.8 ^c^		14.1
	4	49.2 ± 7.9 ^c^		16.1
	5	52.9 ± 8.6 ^d^		16.3
*L. paracasei* in MRS broth	0	42.9 ± 4.8 ^a^	19.1 ± 6.1 ^c^	11.2
	1	45.8 ± 4.9 ^a^		11.0
	2	48.8 ± 5.8 ^b^		11.9
	3	49.5 ± 6.6 ^b^		13.3
	4	50.3 ± 6.7 ^bd^		13.3
	5	51.3 ± 8.3 ^d^		16.1

## Data Availability

Not applicable.

## Supplementary Materials

The following supporting information can be downloaded at: https://www.mdpi.com/article/10.3390/mi13122067/s1, Figure S1: Bacterial adherence to mineral oil for live and dead cells at different mineral oil values, Figure S2: Interfacial tension of bacterial suspension at different concentration against mineral oil, Figure S3: Changes in interfacial tension with the addition of samples containing live and dead cells at different ratio, Figure S4: The zeta potential values of bacterial cells.
