# Supplementary material for: The Effect of Bacteria on the Stability of Microfluidic-Generated Water-in-Oil Droplet
Source: Micromachines (Basel). 2022 Nov 25;13(12):2067. doi: 10.3390/mi13122067 (PMC9785555; doi:10.3390/mi13122067)
Supplement: Supplementary file 1 [file micromachines-13-02067-s001.zip › micromachines-1840352-supplementary.pdf]

The effect of bacteria on the stability of microfluidic-generated water-in-oil droplet

Nur Suaidah Mohd Isa[1,2], Hani El Kadri[1], Daniele Vigolo[1,3,4*], Konstantinos Gkatzionis[1,5*]

[1]School of Chemical Engineering, University of Birmingham, Birmingham B15 2TT, UK
[2] Faculty of Fisheries and Food Science, Universiti Malaysia Terengganu, Kuala Terengganu 21030, Terengganu, Malaysia

[3] School of Biomedical Engineering, The University of Sydney, Sydney, NSW 2006, Australia

[4] The University of Sydney Nano Institute, The University of Sydney, Sydney, NSW 2006, Australia

[5] Department of Food Science and Nutrition, University of the Aegean, Metropolite Ioakeim 2, 81400 Myrina, Lemnos, Greece

*Corresponding authors: daniele.vigolo@sydney.edu.au (D.V.); kgkatzionis@aegean.gr (K.G.)

Supplementary information

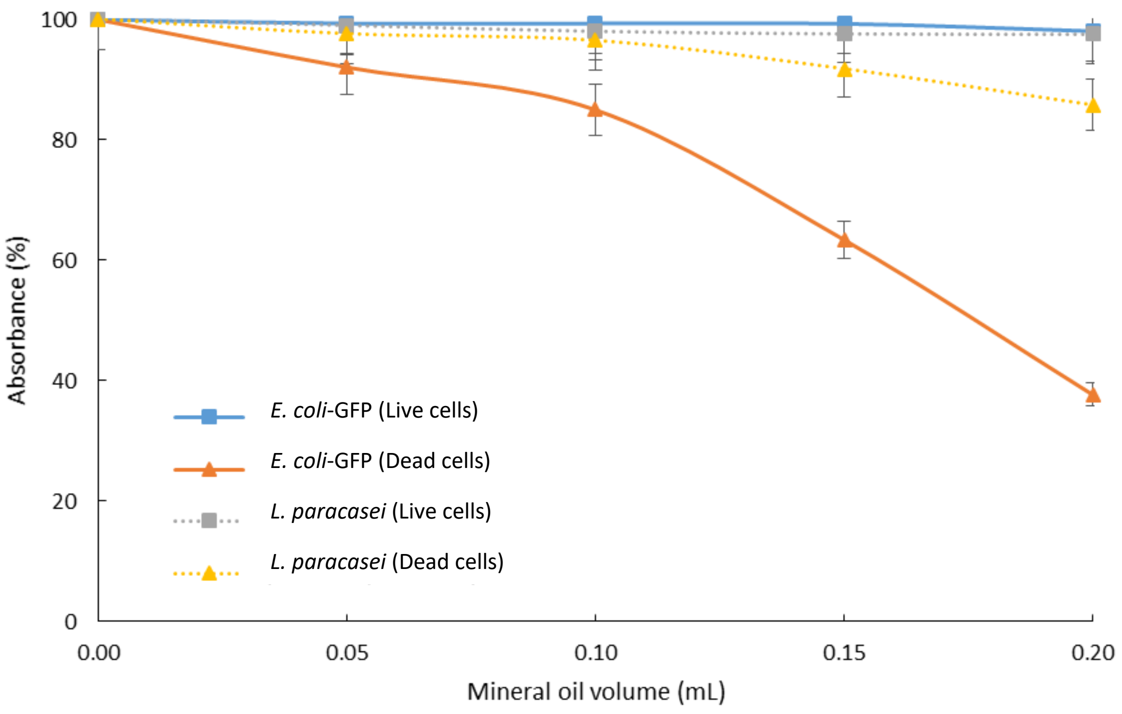

Figure S1. Bacterial adherence to mineral oil for live and dead cells at different mineral oil volume. The absorbance of bacterial suspension taken from each sample was measured against mineral oil volume. Bar represents mean ± SEM for 3 independent experiments (N=3).

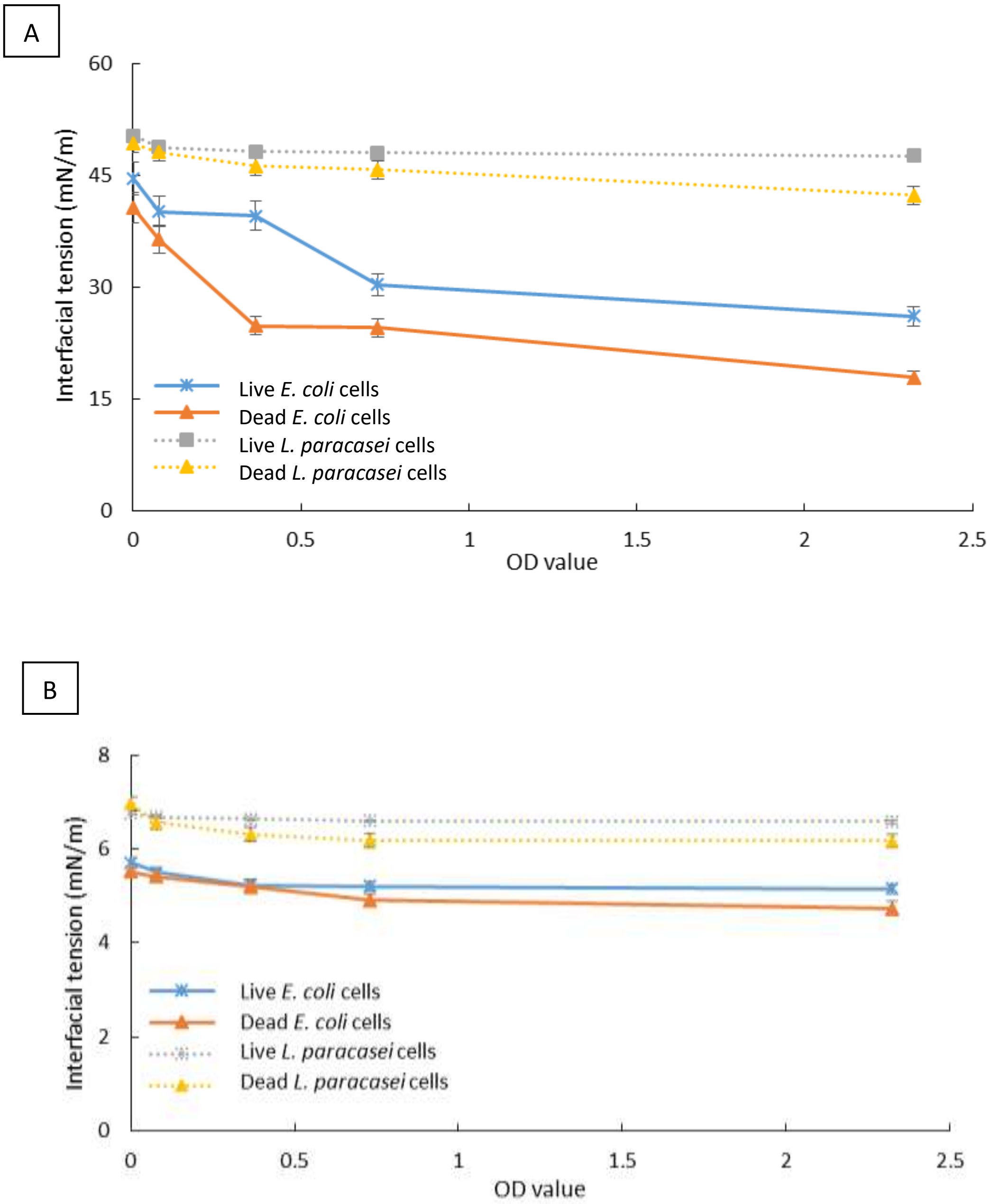

Figure S2. Interfacial tension of bacterial suspension at different concentration against mineral oil with (A) samples without PGPR in the oil phase (B) samples with PGPR in the oil phase. Bar represents mean ± SEM from 3 independent experiments.

A

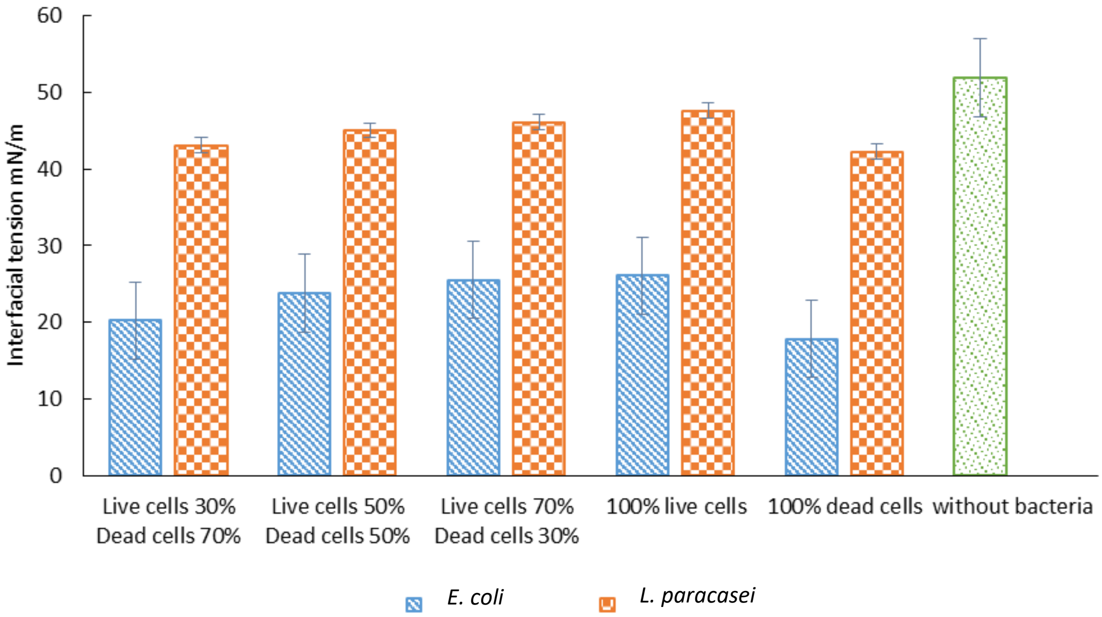

B

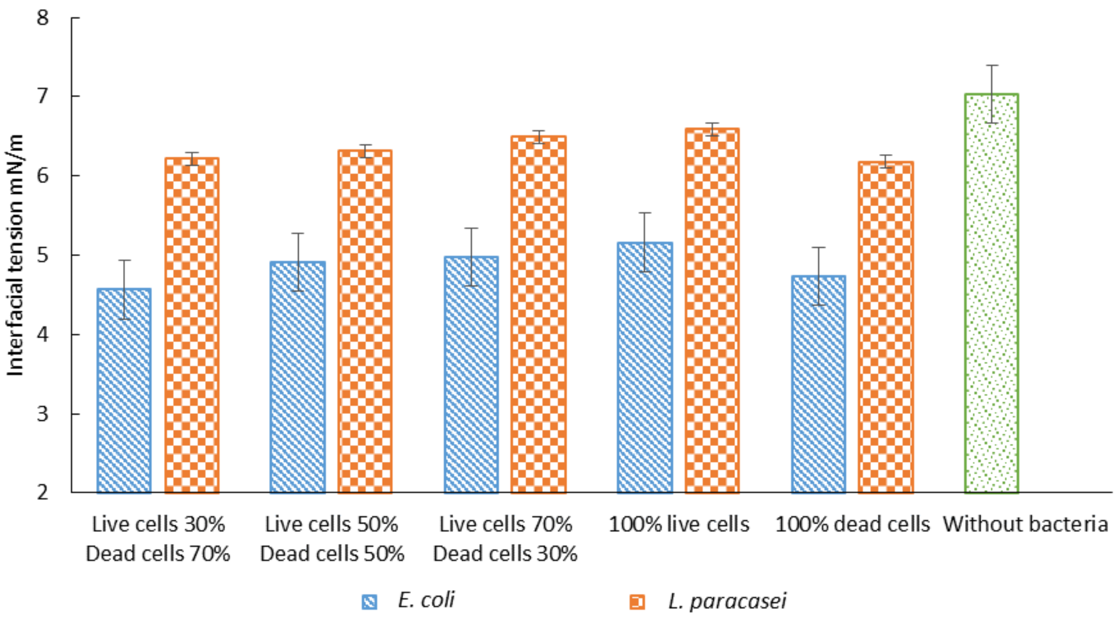

Figure S3. Changes in interfacial tension with the addition of samples containing live and dead cells at a different ratio with (A) without PGPR in the oil phase (B) with PGPR in the oil phase. Bars represent mean ± SEM taken from 3 independent experiments (N=3).

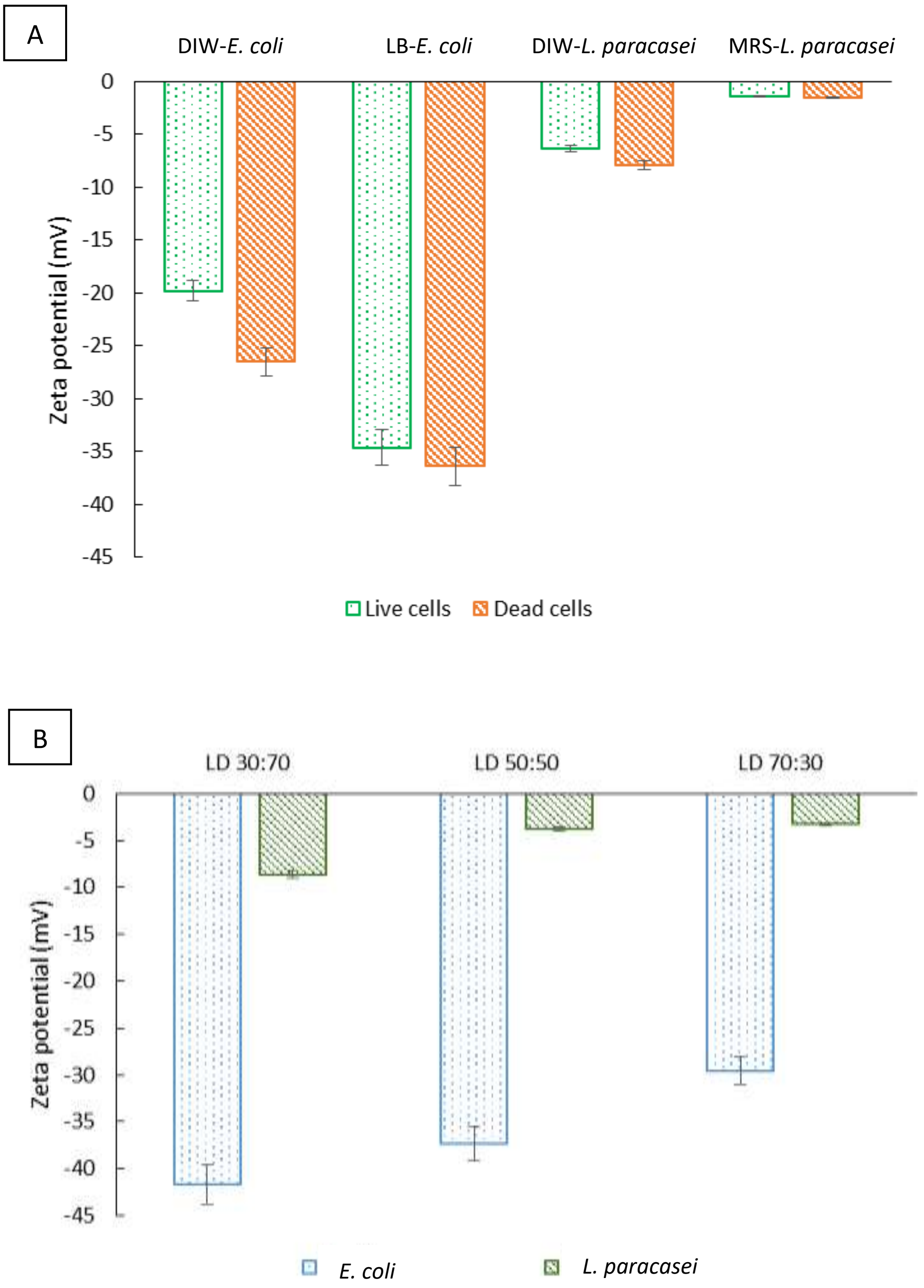

Figure S4. The zeta potential values of bacterial cells for (A) live or dead cells suspended in DIW or with nutrient, (B) mixed samples with Live:Dead cell ratio (L:D) of 30:70, 50:50 and 70:30. Bars represent mean ± SEM taken from 3 independent experiments (N=3).
